# Corrigendum: The Conservation of VIT1-Dependent Iron Distribution in Seeds

**DOI:** 10.3389/fpls.2020.01101

**Published:** 2020-07-17

**Authors:** Seckin Eroglu, Nur Karaca, Katarina Vogel-Mikus, Anja Kavčič, Ertugrul Filiz, Bahattin Tanyolac

**Affiliations:** ^1^Department of Genetics and Bioengineering, Izmir University of Economics, Izmir, Turkey; ^2^Department of Bioengineering, Ege University, Izmir, Turkey; ^3^Department of Biology, University of Ljubljana, Ljubljana, Slovenia; ^4^Jozef Stefan Institute, Ljubljana, Slovenia; ^5^Department of Crop and Animal Production, Cilimli Vocational School, Duzce University, Duzce, Turkey

**Keywords:** biofortification, seed, iron, metal, vit1, plastid, synchrotron, homeostasis

## Missing Funding

In the original article, we neglected to include the funder “The research leading to this result has been supported by the project CALIPSOplus under Grant Agreement 730872 from the EU Framework Programme for Research and Innovation HORIZON 2020.” The authors apologize for this error and state that this does not change the scientific conclusions of the article in any way. The original article has been updated.

